# The Bangor Voice Matching Test: A standardized test for the assessment of voice perception ability

**DOI:** 10.3758/s13428-017-0985-4

**Published:** 2017-11-09

**Authors:** Constanze Mühl, Orla Sheil, Lina Jarutytė, Patricia E. G. Bestelmeyer

**Affiliations:** 1 School of Psychology, Bangor University, Brigantia Building, Penrallt Road, Bangor, Gwynedd LL57 2AS UK; 2 School of Experimental Psychology, University of Bristol, Bristol, BS8 1TU UK

**Keywords:** Voice identity perception, Individual differences, Phonagnosia

## Abstract

Recognising the identity of conspecifics is an important yet highly variable skill. Approximately 2 % of the population suffers from a socially debilitating deficit in face recognition. More recently the existence of a similar deficit in voice perception has emerged (phonagnosia). Face perception tests have been readily available for years, advancing our understanding of underlying mechanisms in face perception. In contrast, voice perception has received less attention, and the construction of standardized voice perception tests has been neglected. Here we report the construction of the first standardized test for voice perception ability. Participants make a same/different identity decision after hearing two voice samples. Item Response Theory guided item selection to ensure the test discriminates between a range of abilities. The test provides a starting point for the systematic exploration of the cognitive and neural mechanisms underlying voice perception. With a high test-retest reliability (*r*=.86) and short assessment duration (~10 min) this test examines individual abilities reliably and quickly and therefore also has potential for use in developmental and neuropsychological populations.

**Electronic supplementary material:**

The online version of this article (10.3758/s13428-017-0985-4) contains supplementary material, which is available to authorized users.

## Introduction

Research in the field of person perception has focused on how we recognize and judge individuals based on their visual characteristics. Early descriptions of face recognition deficits reach back to the 19th century (Quaglino & Borelli, 1867, translated by Della Sala & Young, 2003), and for neurotypical individuals, a remarkable variability in face perception ability has been reported (e.g., Burton, White, & McNeill, 2010; Duchaine & Nakayama, 2006; Huang et al., 2014). Many face tests involve matching pictures of faces to a given identity. Others rely on identification of previously studied faces in novel images, thereby assessing face *memory* in addition to more basic face perception abilities.

Earlier face tests, such as the Benton Facial Recognition Test (Benton & van Allen, 1968) or Warrington’s Short Recognition Memory Test for Faces (1996), were aimed towards use with brain-lesioned patients. Accuracy for healthy adults on these tests was between 84.1 % and 90.4 %, respectively (Benton, Hamsher, Varney, & Spreen, 1983; Warrington, 1996). Interpretation of the test scores is problematic, however, as stimuli in both tests include non-facial information (e.g., visible hairlines, clothing) that can be used to match or recognize target faces correctly without the use of facial information (Duchaine & Nakayama, 2004; Duchaine & Weidenfeld, 2003).

More recent tests address these issues. Among those tests are the Cambridge Face Memory Test (Duchaine & Nakayama, 2006) with a normal distribution of scores and mean performance of 74.2 % (Wilmer et al., 2012) and the Glasgow Face Matching Test (GFMT; Burton et al., 2010). Both tests assess individual face perception in the general population. Our voice-matching test is based on Burton et al.’s (2010) GFMT. Briefly, the GFMT requires participants to make a same/different identity judgment on picture pairs of unfamiliar faces. Half of the items depict two different photos of the same person and the other half consists of photos of different identities. Based on this initial long version (168 items), a test with only 40 items was later constructed. Average performance for healthy adults was 89.9 % (long GFMT) and 81.3 % (short GFMT), with high interindividual variability (Burton et al., 2010). To ascertain that this was not due to individual differences in more general visual abilities, Burton and colleagues included assessments of face memory abilities, visual matching of objects, and visual short-term memory. The GFMT correlated only weakly with face memory abilities and moderately with object-matching abilities. This points to the GFMT measuring a distinct ability rather than a component of general visual abilities or face memory.

While the face is an important visual object for social evaluation, humans also reliably judge individuals based on the sound of their voice (e.g., Bestelmeyer, Belin, & Ladd, 2015; Bestelmeyer et al., 2012; Bestelmeyer, Rouger, DeBruine, & Belin; 2010; Bruckert et al., 2010; Hughes, Dispenza, & Gallup, 2004; Hughes, Harrison, & Gallup, 2002; McAleer, Todorov, & Belin, 2014; O’Connor, Re, & Feinberg, 2011; Vukovic et al., 2011). In fact, similar neural and cognitive mechanisms have been proposed for both face and voice perception (Belin, Fecteau, & Bédard, 2004; Yovel & Belin, 2013). Despite these similarities and the apparent relevance of both modalities in identity perception, the bulk of available literature focuses on faces rather than voices (Blank, Wieland, & von Kriegstein, 2014; Gainotti, 2014). This is ultimately reflected in a lack of validated voice perception tests and currently prevents a methodical comparison of face and voice perception abilities and their possible interactions.

A small literature exists on voice recognition deficits known as phonagnosia. The assessment typically consists of in-house developed tests with voice samples of previously unfamiliar (Roswandowitz et al., 2014), familiar (Peretz et al., 1994, Shilowich & Biederman, 2016), or unfamiliar and famous speakers (van Lancker, Kreiman, & Cummings, 1989), or researchers report impaired perception of more general auditory abilities like perception of prosody or melody (Peretz et al., 1994). For example, Shilowich and Biederman (2016) used an online survey to estimate the voice recognition ability for speech samples of well-known American celebrities in a large sample of 730 participants. Their aim was to determine what percentage of the population, assuming a normal distribution of voice recognition abilities, are likely to meet the criteria for developmental phonagnosia. While Shilowich and Biederman (2016) tried to account for the effect of voice familiarity by basing their analysis on an individually calculated residual, there currently is no test that assesses voice perception abilities per se, using unfamiliar voices independently of speech content.

One recent test, however, has reported a tool for quantifying memory ability for unfamiliar voices (Aglieri et al., 2016). This test, the Glasgow Voice Memory Test, first involves participants to listen to three repetitions of the same vowel sound “ah” produced by eight different speakers. Participants are then tested on 16 voices (eight new), and are required to make an old/new judgment for each voice. In order to assess a voice specific deficit, an otherwise identical second part of the test features bell sounds. Bell sounds were typically recognized more easily (*M* = 85.6 %) than voices (*M* = 78.8 %), making a direct comparison between categories more difficult. The memory component of the test performance may be driven by more general auditory working memory abilities rather than the ability to extract specific voice information alone. The test is, however, very short and easy to administer with a moderate test-retest reliability (*r* = 0.38) for the old/new categorization of voices.

A general difficulty in test construction, particularly in cognitive assessment, is to establish an adequate item pool for the measurement of different perception abilities that assesses a *range of abilities* as well as *discriminates* accurately between ability levels. This problem can be addressed with Item Response Theory (IRT). IRT is a test theory that was first established to address shortcomings of the classical test theory, such as sample dependency of classical tests (Embretson & Reise, 2000; Hambleton & van der Linden, 1982). At first, IRT concepts were mostly applied in educational contexts before eventually extending into the domains of psychopathology and personality psychology, e.g., to assess intelligence (Embretson & Reise, 2000; Reise & Revicki, 2015). Rather than providing models based on test scores, IRT aims to describe the items that make up psychological tests (Hambleton & van der Linden, 1982). As such, IRT provides the tools to choose test items that cover a range of difficulties and discriminate between individuals of different ability levels. This knowledge can also be used to shorten already established tests by eliminating inefficient or unnecessary items. Item selection reduces testing time, facilitates test administration, and allows for better application in demanding assessment settings (e.g., clinical, developmental). While a number of tests mentioned above provide such short versions, it is often unclear or even unstated how item selection took place (e.g., short version of Warrington’s Recognition Memory Test). In other instances, item selection did not follow the analysis of specific item characteristics. For example, highest error rates on items in the long version of the Glasgow Face Matching Test sample were seen as an indicator of item difficulty and thus guided item selection for the short version. Yet it might still be that these difficult items do not discriminate appropriately between individuals who vary in their ability levels.

The aim of our work was therefore to introduce a voice-matching test suitable to measure a wide range of voice perception abilities. The final, short test version includes highly discriminating items across a wide range of ability levels. Our test involves listening to two syllabic utterances per trial followed by a requirement to make a same/different speaker judgment. Phase 1, described in the next section, consists of the results with the initial item pool (288 items) as well as the outcomes of a subsequent IRT analysis to identify the most discriminating items which form the final version of the Bangor Voice Matching Test (80 items). In a new sample of participants (Phase 2; described below), we then report results of our shortened, final voice-matching test and its relationship with a variety of other abilities to demonstrate that voice perception is a unique, high-level ability that does not merely require more general auditory abilities or auditory working memory. To this end our test battery incorporated the Music Perception Skills test (Law & Zentner, 2012) assessing more basic auditory perception skills (e.g., pitch, rhythm) and a digit span test for auditory working memory. Here we predicted small to moderate positive correlations with our voice perception test given that voice matching will have to rely, to some extent, on basic auditory perception and working memory abilities. Additionally, we included the Glasgow Face Matching Test (Burton et al., 2010) in our test battery for which we expected a positive relationship between our test and this face test, given their similar task demands and the parallels reported between voice and face perception. We also administered Aglieri et al.’s (2016) test to examine the relationship between this memory test for unfamiliar voices and our test of more foundational voice perception ability.

## Phase 1: Initial item pool and item selection for the Bangor Voice Matching Test

### Phase 1: Materials and methods

#### Participants

A total of 457 adults (135 male) were recruited from the student and general population. Volunteers participated in exchange for £5 or course credit. Mean age was 22.47 years (*SD* = 7.27). Sixty-eight participants (22 male) took part in a re-test session. The experiment was approved by the School of Psychology’s Ethics committee at Bangor University.

#### Stimuli

Sustained vowels, consonant-vowel-consonant (CVC; had, hed, hid, hod, hood, hud, hide), vowel-consonant-vowel syllables (VCV; aba, aga, ada, ibi, igi, idi, ubu, udu, ugu), and two short paragraphs of text were recorded from several hundred female and male British-English native speakers in a sound attenuated booth. Sounds were recorded using Audacity 2.0.3 (16-bit, 44.1 kHz sampling rate, mono). All speakers were undergraduate students between 18 and 28 years of age. Speakers with a pronounced regional accent or vocal health issues were excluded, which left us with 149 male and 182 female speakers. Baumann and Belin (2010) have shown that a two-dimensional “voice space” between F0 and f1 is sufficient to represent speaker similarity (see also Latinus, McAleer, Bestelmeyer, & Belin, 2013). Within this space, voices that are closer in distance will also be perceived to be more similar (i.e., will be harder to differentiate) than voices that are further apart. We therefore measured F0 and f1 in the stable portion of the sustained vowel /e/ for all voices (after normalization for energy (root mean square)). We then computed the distance between each voice and every other voice separately for male and female voices using the Pythagoras Theorem. The distances for the male and female voices were separately min-max transformed due to male voices generally having shorter distances than female voices. For each sex we selected 40 voice pairs with a distance less than .12, 20 voice pairs with a distance between .22 and .27, and 12 voice pairs with distances greater than .4. We selected voice pairs on this basis to ensure that the items differed in how easily they could be differentiated even before the IRT analysis. As expected we found a correlation between the distance between voice pairs and the percent correct categorization across our full sample of 457 participants (*r* = .39; *p* < .001).

The initial item pool therefore consisted of 288 items (or speaker pairs), 144 items for each speaker sex, which was presented blocked. Half of each block consisted of same-identity pairs and half of different-identity pairs. The two syllable types presented per pair were never the same. Equal numbers of voice pairs per sex (n = 36) consisted of VCV-VCV, CVC-CVC, VCV-CVC, and CVC-VCV pairs. We used 16 instances of each CVC and 21 instances of each VCV syllable (except “hid”; n = 18). Block order and trials within each block were randomized for each participant. All test stimuli were root-mean square normalized and edited in Adobe Audition to start with onset of phonation and end with the offset of phonation (mean duration = .51s; SD = .11).

#### Procedure

Up to three participants were tested simultaneously on separate computers. We used Psychtoolbox-3 (Brainard, 1997; Kleiner, Brainard, & Pelli, 2007) for Matlab (2013a) to present the stimulus pairs. Stimuli were presented binaurally via Beyerdynamic DT770 Pro headphones (250 Ω). The display for each trial (or each test item) consisted of two red speaker icons in the top left- and top right-hand sides of the screen and two labels in the bottom left and right-hand sides stating “Same Speaker” or “Different Speakers” (see Supplementary Fig. S1 for an illustration of the trial structure). Initial verbal and on-screen instructions informed the participant that voice samples will be played following a mouse-click on each icon and that the same/different judgment was made by clicking on one of the two labels underneath. Participants were able to listen to the voice sample multiple times by clicking on the same icon. Between trials, a fixation cross would appear for 800 ms. Overall trial durations were self-paced but participants were instructed not to overthink their choice. Testing lasted approximately 40 min.

### Phase 1: Results

#### Item analysis: Selection of most discriminating items for the final, short version of the test

Item analysis was conducted in R (version 3.0.1), using the ltm package for latent trait models in R (Rizopoulos, 2006). Same and different items were analyzed separately to account for the difference in correctly identifying an item pair of the same identity versus correctly distinguishing two voice samples as different. Several IRT models are available to distinguish between different item parameters relevant for test construction. Three models can be used for binary items such as same/different judgments: the Rasch model, the two-parameter logistic model, and the three-parameter logistic model (Rizopoulos, 2006). The Rasch model assumes that items only differ in their difficulty. The two-parameter logistic model includes an additional discrimination parameter. This parameter describes how well items differentiate between subjects with different ability levels. The three-parameter logistic model adds a third parameter for guessing the right response. Each model provides an estimation of its model-specific item parameters. These can then be used to guide item selection.

Model comparison using the Akaike’s information criterion for all three models revealed the two-parameter logistic model to be best suited for item parameter estimation of the dataset of our initial item pool of 288 items (Table 1). Item parameters under the two-parameter logistic model for all 288 items are included in Supplementary Tables S1 and S2, as are test information curves for both same and different items (Figs. S2 and S3 of the Supplementary Online Material). Items with discrimination of at least .80 were then identified. From those, 80 items (40 male voices) were selected to form the final test version of the Bangor Voice Matching Test. Items were selected on the basis of their discrimination scores (> .80) and their difficulty scores in order to span a wide range of ability levels (difficulty scores between -4.81 to 0.54). The selected items are highlighted in gray in Table S1 and S2 of the Supplementary Online Material. The two-parameter logistic model was fitted on this short-test version again. Item parameters as well as test information curves for same and different items of the short Bangor Voice Matching Test are given in Supplementary Tables S3 and S4 and Supplementary Figs. S4 and S5, respectively. Items with relatively lower discrimination (< 1.00) were kept to ascertain measurement of a wider range of voice-matching abilities. The aim of this item selection was to shorten the test duration considerably (10 min compared to the initial 45 min) while ensuring that the test still covers a wide range of possible ability levels. The final, short version of the Bangor Voice Matching Test is available upon request from the corresponding author.

**Table 1:** AIC (Akaike Information Criterion) values for model comparisons for same and different items

	Items – same identity	p-value	Items – different identities	p-value
Rasch	46944.5		59421.2	
Two-parameter	46665.0	< .001	58962.3	< .001
Three-parameter	46809.0	.484	59080.6	.071

#### Overall performance

On average, participants correctly categorized 75.99 % (*SD* = 5.55) of the voice pairs. Scores were slightly negatively skewed (skewness = -1.00). Test-retest correlation for the initial item pool was high (*r* = .80). Re-analysis of the items comprising the final shortened test on this sample revealed a mean accuracy of 85.51 % (*SD* = 8.58). Again, scores were negatively skewed (skewness = -1.35). Test-retest correlation for this short version was high (*r* = .86). Internal reliability analysis of the shortened test showed a Cronbach’s α of .75, indicating acceptable internal reliability for the BVMT (Ponterotto & Ruckdeschel, 2007). Additionally, we assessed the overall time taken to complete the test (*M =* 8:36 min, *SD* = 1:44). Test accuracy did not correlate with overall test duration, *r* = - .09, *p* = .271.

## Phase 2: Test validation (short, final version of the Bangor Voice Matching Test)

### Phase 2: Materials and methods

#### Participants

Three tests that were predicted to co-vary with our test were assessed in a new sample of 151 native-English speakers. Two participants had to be excluded due to a temporary internet fault. The remaining 149 participants (36 male; mean age = 20.49; *SD* = 4.60) were included in subsequent analyses. As testing had already started by the time Aglieri et al.’s (2016) voice memory task was published, the Glasgow Voice Memory Test could only be included for a subsample of our participants (*n* = 128). The School of Psychology’s Ethics committee at Bangor University approved the experiment. All participants were young adults who completed the experiment in exchange for £5 or course credit.

#### Stimuli and materials

The short version of our voice-matching test follows the same structure as described in Phase 1, but instead consists of 80 items (speaker pairs) presented in two blocks (40 items with male speakers, 40 items with female speakers). One half of each block presents same-identity items, the other half different-identity items. We prioritized item selection based on difficulty and discrimination parameters, as obtained via the IRT analysis, rather than syllable type. Nevertheless, a minimum of eight syllable pairs of each type (CVC-CVC, VCV-VCV, VCV-CVC, or CVC-VCV) were included for each speaker sex (mean duration = .50s; SD = .10). Block order and trials within each block were randomized for each participant. In addition to the Bangor Voice Matching Test, we administered the following tests:

Glasgow Face Matching Test: After initial instructions, participants completed 40 trials of same/different identity judgments of face pairs (two faces next to each other on a gray background). Judgments were made by clicking on one of two labels (same or different identity) located underneath the face display. Each item was followed by a blank gray screen for 800 ms. Testing was self-paced. The test and normative data are downloadable online at http://www.facevar.com/downloads.

Profile of Music Perception Skills: The Profile of Music Perception Skills test is a standardized online test to assess musical perception skills in the general population. It measures multiple facets of auditory perception. Several test versions that differ in composition and length are available online at https://www.uibk.ac.at/psychologie/fachbereiche/pdd/personality_assessment/proms/take-the-test/.

We included the brief test version (duration: ~30 min), which comprises assessments of melody, tempo, tuning, and rhythm perception. Trials consist of two standard melodies (inter-stimulus interval: 1.5 s) and a third comparison stimulus (2.5 s after the standard stimulus). Participants have to decide whether the third one is identical to the first two or whether it differs. Each block consists of 18 trials (nine same) and is preceded by on-screen instructions. Participants receive their results on-screen upon completion.

##### Digit span

The digit span test requires participants to listen to a list of numbers which they have to recall in the correct order (test protocol as used by Della Sala, Foley, Beschin, Allerhand, & Logie, 2010). List length increases with successful completion of each set of numbers. In the present study, participants were presented with a three-digit list first. The experimenter read out one number at a time (one digit per second) and the participant had to repeat the number immediately afterwards. Six numbers for each list length were presented, and performance was scored on a sheet of paper. If the participant correctly recalled at least five out of six numbers, the next list (here: four-digit numbers) was started, and so on. Final digit span score represents the maximum list length that was correctly recalled (five out of six numbers). The test protocol is available online at http://www.ppls.ed.ac.uk/psychology/people/sergio-della-sala#tests *.*

##### Glasgow Voice Memory Test

Following an instruction screen, during the study phase of this test, participants listened to eight speakers (four male) voicing the vowel /a/ three times. This was immediately followed by the test phase during which 16 voices (eight male) articulated the same vowel. For each of these vowels, participants had to indicate via a key press whether the voice was old (presented during the study phase) or new. The second part of the test was identical but featured bell sounds instead of voices. The test is available online at http://experiments.psy.gla.ac.uk/.

#### Procedure

We administered the following tests in randomized order: our voice-matching test, the short GFMT to assess face-matching abilities (Burton et al., 2010), the internet-based Profile of Music Perception Skills to assess general auditory abilities (Law & Zentner, 2012), and a digit span test for auditory working memory (Della Sala et al., 2010). The Glasgow Voice Memory Test (Aglieri et al., 2016) was also assessed to compare performance on both standardized voice ability tests. Overall, testing took approximately 60 min. Up to two participants were tested simultaneously. Face and voice tests were implemented in Matlab (2013a) and Psychtoolbox-3 (Brainard, 1997; Kleiner et al., 2007).

#### Phase 2: Results

**Fig. 1:**
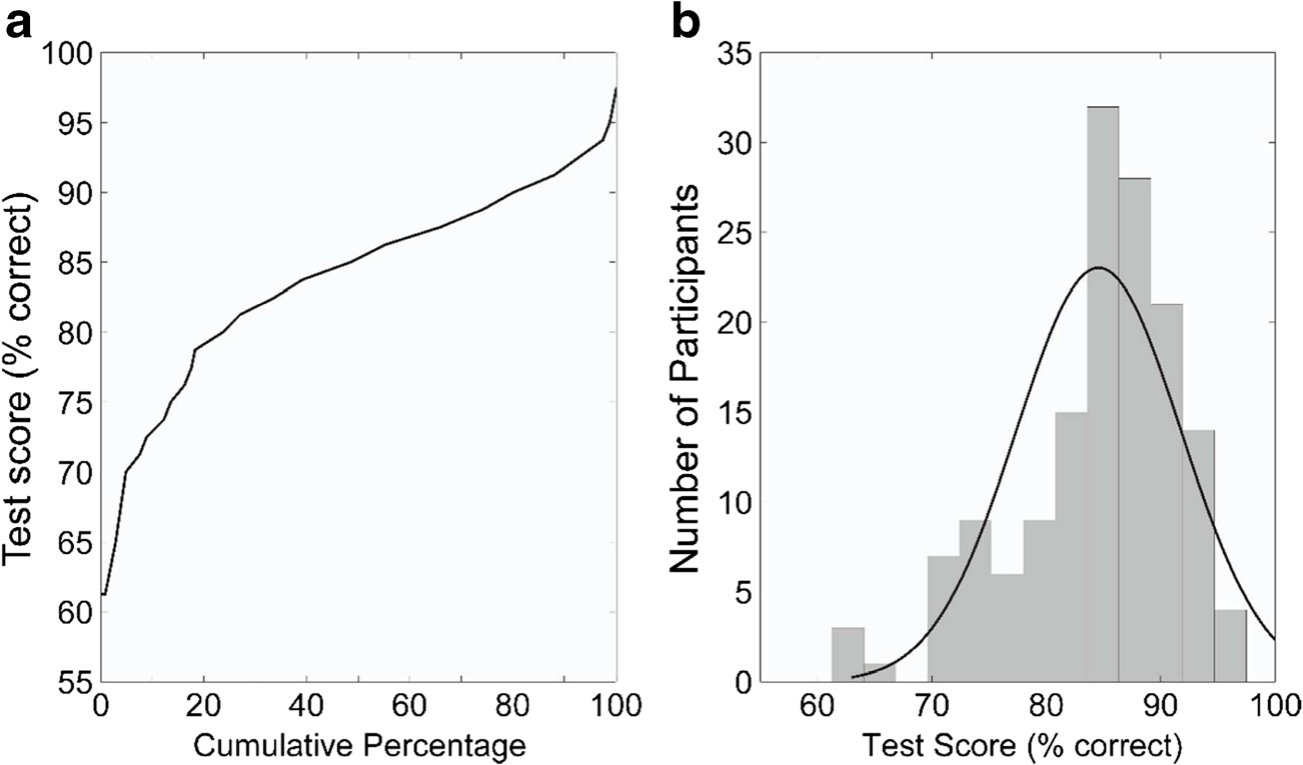
(**A**) Cumulative frequencies of test scores (% correct) and (**B**) test score distribution (% correct) for the Bangor Voice Matching Test

Figure 1A shows the cumulative frequency of test scores (percentage correct) for the Bangor Voice Matching Test while Fig. 1B shows the distribution of test scores (percentage correct). Pearson correlations between the percentage scores of the short Bangor Voice Matching Test and all possible covariates were then calculated using SPSS (version 22). We performed five correlations in total; three between our voice test and the covariate tests (face, music and digit span test) and two between our voice test and the Glasgow Voice Memory Test (separately for voices and bells). Curtin and Schulz (1998) point out that the risk of type 1 errors increases with additional correlations, even for small numbers of additional comparisons. We therefore applied a Bonferroni correction to control for multiple comparisons, which led to an adjusted p-value of .01.

Mean performance on our test was 84.57 % (*SD =* 7.20*,* range: 61.25–97.50 %*).* Descriptive statistics for the three covariate tests as well as their correlations with the Bangor Voice Matching Test can be seen in Table 2. Performance on our Bangor Voice Matching Test correlated weakly with both face matching performance on the Glasgow Face Matching Test and auditory working memory as measured by the Digit Span test. There was a moderate positive relationship between the Bangor Voice Matching Test and general auditory abilities, as assessed via the Profile of Music Perception Skills. All correlations were significant at *p* < .01. Test validation also included the Glasgow Voice Memory Test for vocal and non-vocal memory performance. The relationship between both voice tests was only weak, and similar in strength for correlations of our voice-matching test with both subtests of the Glasgow Voice Memory Test (voice memory: *r* = .23; bell memory: *r* = .25). This suggests that both the Bangor Voice Matching Test and the Glasgow Voice Memory Test might assess diverging parts of voice perception. All other correlations between the Glasgow Voice Memory Test (voice memory) and possible covariates of auditory perception (Music Perception Skills, Digit Span) were also weak and did not reach significance (Music Perception Skills: *r* = .14; Digit Span: r = -.01; all p > .125). A table of all inter-correlations between both voice tests and the covariates can be found in Supplementary Table S5.

**Table 2.** Descriptives and correlations (Pearson’s r) of covariates with the Bangor Voice Matching Test

	Glasgow Face Matching Test (%)	Profile of Music Perception Skills (%)	Digit Span (total items recalled)
Mean	78.42	59.93	5.09
SD	10.89	9.94	1.07
Correlations with BVMT (p-value)	.24 (.004)	.37 (< .001)	.25 (.003)

Descriptives for Glasgow Face Matching Test and Profile of Music Perception Skills are in percentage correct Mean and SD for Digit Span represent scores, i.e., number of digits held in auditory working memory. Numbers in parentheses represent *p*-values for correlations with the Bangor Voice Matching Test (BVMT)

## Discussion

The primary goal of this study was to create a standardized voice test to reliably assess individual ability levels for voice matching. The test structure follows that of a well-established face test (Glasgow Face Matching Test; Burton et al., 2010). Item selection was based on Item Response Theory to aid the construction of a test that assesses a wide range of ability levels and allows for discrimination between individual performance levels. The test has a high test-retest reliability (r = .86) as well as acceptable internal reliability (α = .75). Simultaneous appraisal of potential covariates showed only weak to moderate correlations with our voice-matching test, supporting the notion that the Bangor Voice Matching Test measures an ability that is distinct from general auditory abilities and auditory working memory. Our test provides a stepping-stone towards further studies exploring the neural and cognitive underpinnings leading to individual differences in voice perception ability.

With a test duration of ~10 min, the Bangor Voice Matching Test is easy and quick to use even within a test battery. This can be particularly important in settings where demands on the participant are high, or environmental factors limit testing time, for example in clinical settings. While for these instances, norm data for patients needs to be established first, the current information can already be used to distinguish different ability levels. The test score is therefore a good indicator for performance levels below average that might call for further investigation. The Bangor Voice Matching Test can therefore help further our understanding of voice perception, its cognitive mechanisms and possible deficits.

Memory demands for the Bangor Voice Matching Test are kept to a minimum by choosing a trial design in which participants can listen to stimuli multiple times, and make their decision immediately following stimulus presentation. Nevertheless, participants still need to hold the voice samples in working memory before deciding whether those are from the same or different speakers. Voice test performance might thus be influenced, to some degree, by auditory working memory ability. A correlation between our voice test and digit span scores, however, showed only a weak relationship. Differences in accuracy on the voice test can therefore not only be attributed to differences in auditory working memory.

Additionally, performance on our voice-matching test cannot solely be attributed to general auditory abilities like rhythm or pitch perception, as measured by the Music Perception Skills test. The correlation between both tests was moderate. While voices as auditory objects consist of such components as pitch, the perception of voices is still distinct from non-vocal sounds (e.g., Leaver & Rauschecker, 2010).

Theoretical models (e.g., Belin et al., 2004) and recent empirical evidence point towards interacting, possibly similar cognitive processes for face and voice perception (e.g., Bestelmeyer et al., 2010; Pye & Bestelmeyer, 2015; Schweinberger, Robertson, & Kaufmann, 2007; Schweinberger, Kloth, & Robertson, 2011; Yovel & Belin, 2013; Zäske, Schweinberger, & Kawahara, 2010). We found a weak correlation between the Bangor Voice Matching Test and the GFMT. This may point towards the existence of interacting face and voice modules. However, neuropsychological evidence also supports the notion of a double dissociation between face and voice perception. In some clinical cases, face perception was disrupted, but not voice perception (van Lancker & Canter, 1982), or vice versa (Neuner & Schweinberger, 2000). Rather than capturing the interaction of face and voice modules, the correlation we found could instead reflect the presence of a common underlying factor of face and voice identity perception, for example intelligence or sociability.

Importantly, even though the task is straightforward and participants can listen to voice samples multiple times before making their decision, performance levels still varied to a substantial degree. Twelve percent of all participants scored 1.5 or more SDs below average. Russell, Duchaine and Nakayama (2009) and Wilmer et al. (2012) suggest that face perception is normally distributed in the population, with the tails of the distribution indicating prosopagnosics and super-recognizers. Given the parallel nature proposed for face and voice perception, it is possible that the same rings true for voice perception. In this case, the Bangor Voice Matching Test can provide a valuable tool to find more individuals whose voice perception abilities, specifically their ability to discriminate and match unfamiliar voices, are below average or possibly even severely impaired.

Developmental phonagnosia, as reported by Garrido et al. (2009) and Herald, Xu, Biederman, Amir, and Shilowich (2014), is the inability to recognize familiar voices. While previous research has shown that impairments in voice recognition are dissociable from impairments in voice discrimination (Neuner & Schweinberger, 2000; van Lancker & Kreiman, 1987; van Lancker et al., 1989), it is possible for both to be affected simultaneously, for example, in participant AS in Roswandowitz and colleagues’ study (2014). In addition to this possibility of co-existing deficits of multiple dissociable voice perception abilities, it is also possible that individuals with a selective impairment of voice-matching ability exist. The Bangor Voice Matching Test can provide a simple tool to identify individuals with such deficits, particularly since the test is especially sensitive for below average ability levels. Note, though, that the norm data reported in this study stems from a sample of young adults. Further investigation of other age groups will be necessary. Implementing the Bangor Voice Matching Test online will facilitate the relatively quick screening of a larger and more diverse sample. We hope that the BVMT will also encourage the development of similar standardized tools probing additional aspects of voice perception such as the ability to determine vocal affect. These additional tests could be beneficial to arrive at a better understanding of the complexities of voice perception. While we assume that the different cues carried by voices (e.g., identity, gender, affect) will initially rely on the perception of similar low-level components, Belin et al.’ (2004) model of voice perception proposes distinct pathways for subsequent higher processing stages. Assessing these other aspects of voice perception might therefore complement the appraisal of someone’s voice-matching ability, and ultimately improve our understanding of the intricate mechanisms underlying voice perception.

Prior to the construction of our voice-matching test, item characteristics such as their difficulty and suitability to judge individual ability levels were not known. To assess them, we used Item Response Theory (IRT). This IRT-driven approach to item selection ensured that the items in the final, short version of the Bangor Voice Matching Test showed adequate item properties in terms of discrimination and range of difficulties. The items for the current voice test were chosen to cover a wide range of ability levels while keeping the discrimination rates satisfactory. However, average or above average ability levels are represented by fewer items that do not cover as wide a range above the average ability level, and their discrimination is not as good as at the lower end of the distribution. To overcome these limitations, a future test version may consider including harder items, for example, by superimposing noise on the stimuli. This method has also been used in the Cambridge Face Memory Test (Duchaine & Nakayama, 2006) to provide more challenging items.

In conclusion, the high test-retest reliability (*r* = .86) of our test and its specificity to voice demonstrate that it is a valuable measurement tool for the systematic exploration of individual differences in voice perception ability. Item selection relied on an IRT-driven approach to ensure that the test discriminates between a wide range of abilities. Thus, our test can be used for a variety of important and novel research questions, e.g., the exploration of similarities and differences in voice and face perception mechanisms or the investigation of the relationship between neural activity in voice-sensitive cortex and behavior. Its short duration and easy administration makes it a potential tool for the investigation of voice perception abilities in under-researched populations such as in children, older adults and individuals with brain lesions.

## Electronic supplementary material

ESM 1 (DOCX 350 kb)
