# Supplementary material for: The Bangor Voice Matching Test: A standardized test for the assessment of voice perception ability
Source: Behav Res Methods. 2017 Nov 9;50(6):2184–92. doi: 10.3758/s13428-017-0985-4 (PMC6267520; doi:10.3758/s13428-017-0985-4)
Supplement: Supplementary file 1 — (DOCX 350 kb) [file 13428_2017_985_MOESM1_ESM.docx]

**Supplementary Online Material**

Figure S1


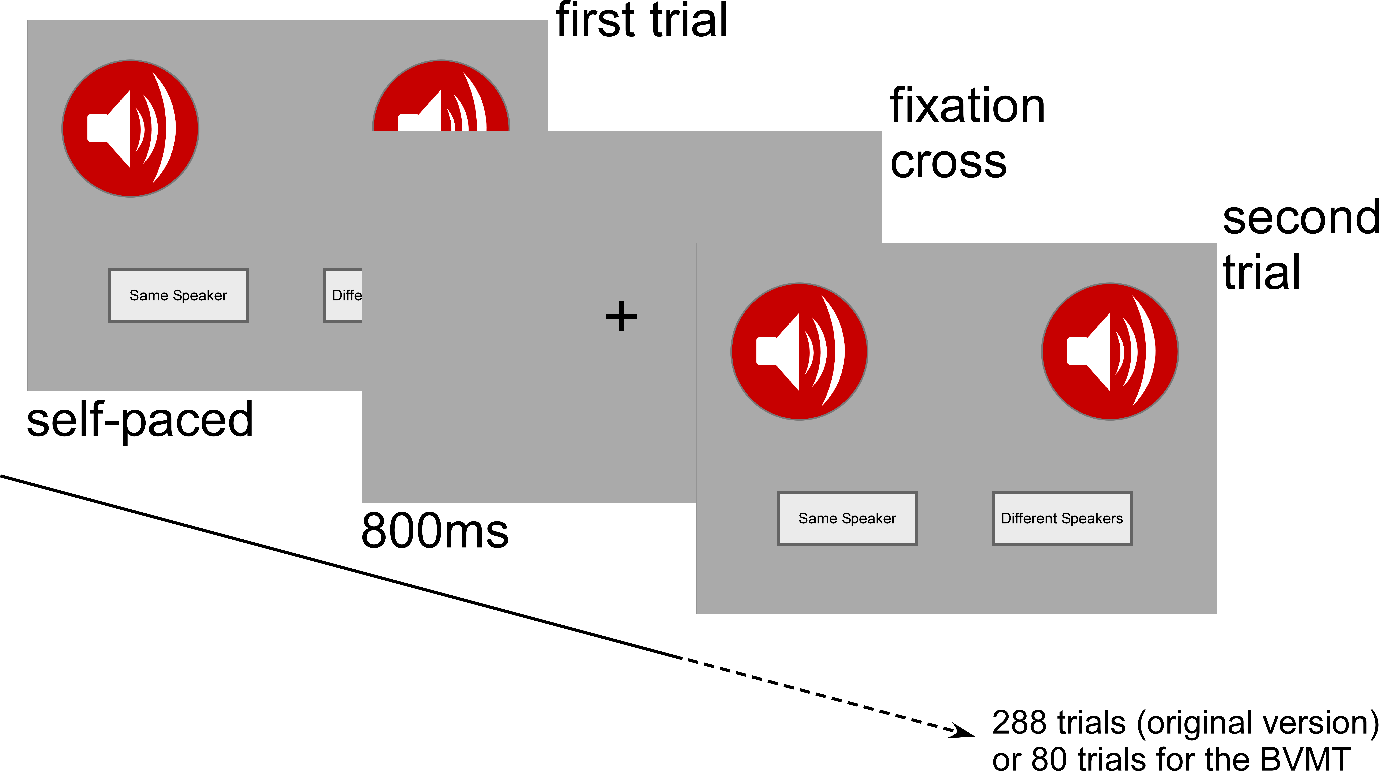


Figure S2

*
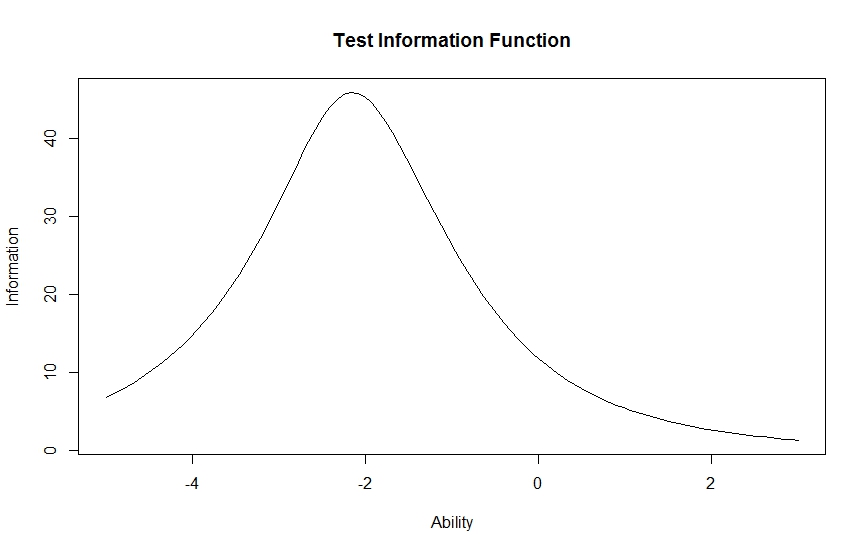
*

Figure S3


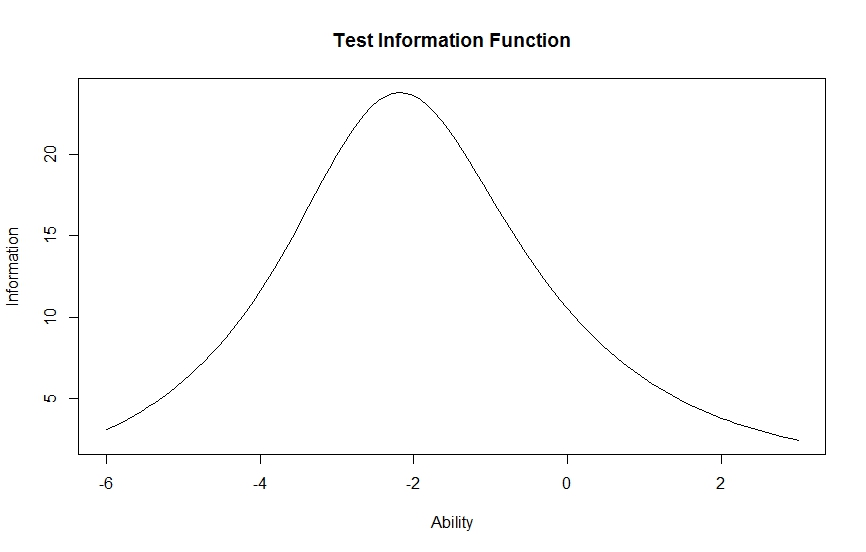


Figure S4

*
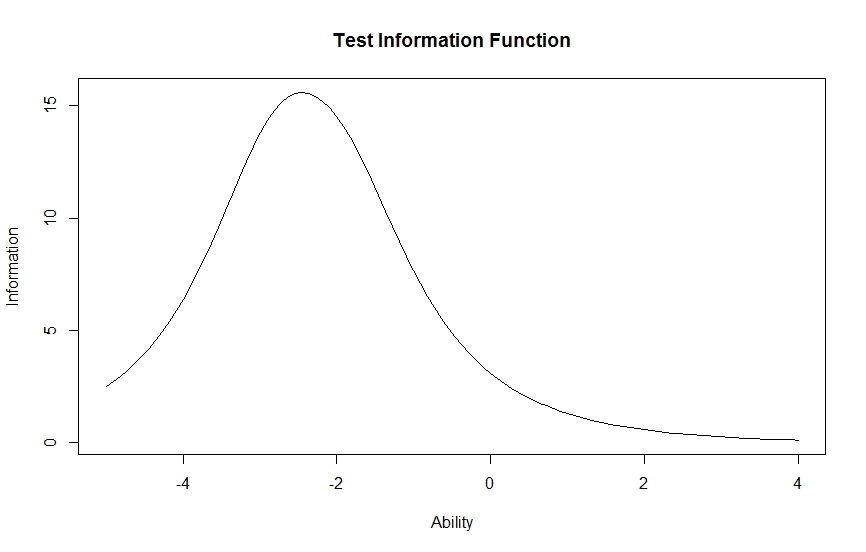
*

Figure S5


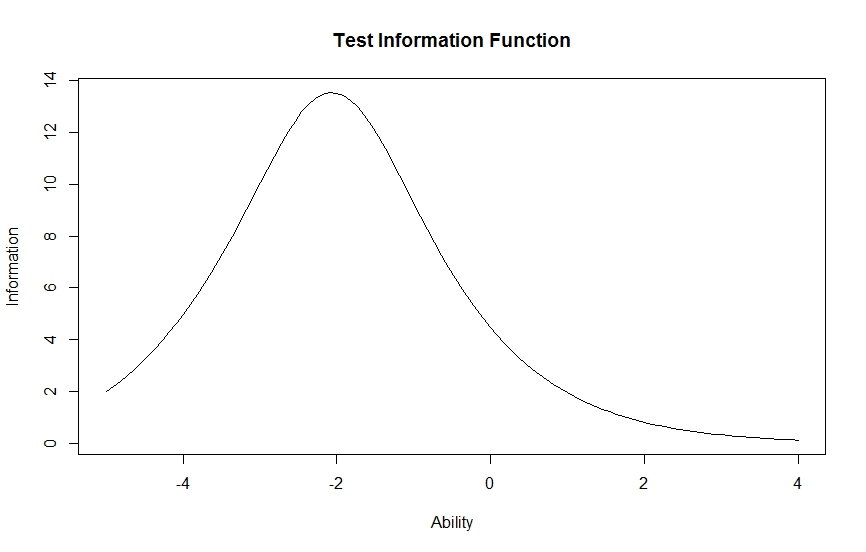


*Figure S1.* Schematic illustration of the trial structure for both versions of the test.

*Figure S2.* Test information curve for items presenting same speaker identity (long version in preparation for the final test).

*Figure S3.* Test information curve for items presenting different speaker identities (long version in preparation for the final test).

*Figure S4.* Test information curve for items presenting same speaker identity of the final, short version of the Bangor Voice Matching Test.

*Figure S5.* Test information curve for items presenting different speaker identities of the final, short version of the Bangor Voice Matching Test.

Table S1

*Item parameters for items presenting same speaker identity (long version in preparation for the final short test version). Item selection for the short test version is highlighted in grey.*

|  | **Difficulty** | **Discrimination** |
| --- | --- | --- |
| Item1 | -2.27 | 1.12 |
| Item2 | -2.15 | 2.12 |
| Item3 | -2.61 | 0.69 |
| Item4 | -2.26 | 1.58 |
| Item5 | -3.56 | 0.67 |
| Item6 | -1.18 | 0.97 |
| Item7 | -2.56 | 1.47 |
| Item8 | -3.42 | 0.61 |
| Item9 | -0.01 | 0.17 |
| Item10 | -0.27 | 0.89 |
| Item11 | 3.94 | 0.35 |
| Item12 | -0.92 | 0.73 |
| Item13 | 0.07 | 0.55 |
| Item14 | -3.08 | 0.60 |
| Item15 | -2.05 | 1.55 |
| Item16 | 0.90 | 0.33 |
| Item17 | -2.43 | 1.05 |
| Item18 | -5.27 | 0.57 |
| Item19 | -2.41 | 1.30 |
| Item20 | -1.99 | 1.21 |
| Item21 | -1.95 | 0.36 |
| Item22 | -2.01 | 0.81 |
| Item23 | -4.50 | 0.98 |
| Item24 | -4.16 | 0.67 |
| Item25 | -2.29 | 1.18 |
| Item26 | -1.90 | 0.95 |
| Item27 | -1.94 | 0.89 |
| Item28 | -1.35 | 0.79 |
| Item29 | -2.08 | 0.96 |
| Item30 | -0.63 | 0.64 |
| Item31 | -2.34 | 0.87 |
| Item32 | -1.83 | 1.53 |
| Item33 | -2.86 | 0.48 |
| Item34 | -1.04 | 0.96 |
| Item35 | -1.63 | 1.22 |
| Item36 | -1.87 | 0.66 |
| Item37 | -2.75 | 1.36 |
| Item38 | -2.25 | 1.83 |
| Item39 | -2.66 | 1.12 |
| Item40 | 0.37 | 0.38 |
| Item41 | -2.58 | 0.88 |
| Item42 | -2.67 | 1.45 |
| Item43 | -1.26 | 0.74 |
| Item44 | -1.90 | 0.82 |
| Item45 | -0.47 | 0.58 |
| Item46 | 1.25 | 0.35 |
| Item47 | -3.12 | 0.87 |
| Item48 | -0.73 | 0.90 |
| Item49 | -1.70 | 1.25 |
| Item50 | -1.66 | 1.93 |
| Item51 | -2.26 | 1.58 |
| Item52 | -2.88 | 0.71 |
| Item53 | -1.79 | 1.05 |
| Item54 | -2.63 | 1.52 |
| Item55 | -2.42 | 1.64 |
| Item56 | 0.54 | 0.91 |
| Item57 | -2.45 | 1.53 |
| Item58 | -2.13 | 1.52 |
| Item59 | -0.17 | 0.54 |
| Item60 | -1.28 | 0.99 |
| Item61 | -3.36 | 1.35 |
| Item62 | -2.34 | 2.52 |
| Item63 | 0.19 | 0.41 |
| Item64 | -3.66 | 0.49 |
| Item65 | -2.78 | 1.06 |
| Item66 | -2.38 | 1.04 |
| Item67 | -3.72 | 0.82 |
| Item68 | -0.85 | 0.60 |
| Item69 | -1.60 | 1.45 |
| Item70 | -3.67 | 0.56 |
| Item71 | -2.45 | 0.60 |
| Item72 | -4.25 | 1.21 |
| Item145 | -2.30 | 1.72 |
| Item146 | -1.70 | 0.82 |
| Item147 | -2.04 | 0.54 |
| Item148 | -2.20 | 1.68 |
| Item149 | -1.84 | 0.80 |
| Item150 | -1.32 | 0.92 |
| Item151 | -1.42 | 0.95 |
| Item152 | -0.01 | 0.39 |
| Item153 | -1.09 | 0.97 |
| Item154 | -0.55 | 0.58 |
| Item155 | -1.33 | 1.23 |
| Item156 | -2.61 | 0.74 |
| Item157 | -3.76 | 0.55 |
| Item158 | -2.07 | 1.13 |
| Item159 | -1.58 | 1.10 |
| Item160 | -2.06 | 1.43 |
| Item161 | -1.41 | 0.91 |
| Item162 | -2.61 | 1.03 |
| Item163 | -2.21 | 1.17 |
| Item164 | -3.37 | 0.35 |
| Item165 | -2.17 | 1.31 |
| Item166 | -2.35 | 1.51 |
| Item167 | -3.77 | 1.13 |
| Item168 | -3.60 | 1.03 |
| Item169 | -1.99 | 1.28 |
| Item170 | -2.83 | 0.77 |
| Item171 | -1.39 | 1.00 |
| Item172 | -2.44 | 1.22 |
| Item173 | -1.77 | 1.13 |
| Item174 | -1.52 | 1.74 |
| Item175 | -4.04 | 0.62 |
| Item176 | -2.30 | 2.36 |
| Item177 | -1.76 | 1.04 |
| Item178 | -1.71 | 1.14 |
| Item179 | -2.08 | 1.72 |
| Item180 | -1.74 | 1.67 |
| Item181 | -1.19 | 0.40 |
| Item182 | -3.29 | 0.88 |
| Item183 | -1.85 | 1.02 |
| Item184 | -2.94 | 0.52 |
| Item185 | -2.89 | 0.99 |
| Item186 | -2.05 | 2.17 |
| Item187 | -3.02 | 0.51 |
| Item188 | -1.85 | 1.13 |
| Item189 | -3.60 | 1.34 |
| Item190 | -2.21 | 1.23 |
| Item191 | -3.00 | 1.09 |
| Item192 | -2.59 | 1.22 |
| Item193 | -1.17 | 1.09 |
| Item194 | -2.20 | 1.31 |
| Item195 | -2.45 | 1.52 |
| Item196 | -3.09 | 0.93 |
| Item197 | -1.44 | 0.42 |
| Item198 | -1.50 | 1.60 |
| Item199 | -2.37 | 2.22 |
| Item200 | -1.23 | 0.88 |
| Item201 | -1.69 | 2.29 |
| Item202 | 0.13 | 0.66 |
| Item203 | -2.05 | 1.44 |
| Item204 | -2.32 | 1.97 |
| Item205 | -5.58 | 0.58 |
| Item206 | 0.08 | 0.82 |
| Item207 | -1.44 | 1.60 |
| Item208 | -2.04 | 1.74 |
| Item209 | -2.08 | 0.75 |
| Item210 | -3.05 | 0.69 |
| Item211 | -2.63 | 1.18 |
| Item212 | -1.93 | 1.32 |
| Item213 | -2.12 | 1.53 |
| Item214 | -1.36 | 1.04 |
| Item215 | -2.14 | 3.42 |
| Item216 | -2.03 | 0.92 |

Table S2

*Item parameters for items presenting different speaker identities (long version in preparation for the final short test version). Item selection for short test version is highlighted in grey.*

|  | Difficulty | Discrimination |
| --- | --- | --- |
| Item73 | -3.36 | 0.95 |
| Item74 | 9.48 | 0.18 |
| Item75 | -1.98 | 0.90 |
| Item76 | -2.50 | 0.59 |
| Item77 | -2.64 | 0.59 |
| Item78 | 11.95 | 0.20 |
| Item79 | -1.83 | 0.69 |
| Item80 | -1.81 | 0.75 |
| Item81 | -2.76 | 0.48 |
| Item82 | -1.01 | 0.91 |
| Item83 | 4.72 | 0.36 |
| Item84 | 4.14 | 0.54 |
| Item85 | -4.36 | 0.38 |
| Item86 | -0.15 | 0.66 |
| Item87 | -2.00 | 0.92 |
| Item88 | 11.02 | 0.24 |
| Item89 | 17.34 | 0.06 |
| Item90 | -17.17 | -0.05 |
| Item91 | 0.18 | 0.54 |
| Item92 | -2.92 | 0.82 |
| Item93 | 0.54 | 0.38 |
| Item94 | -0.75 | 0.42 |
| Item95 | 1.10 | 0.35 |
| Item96 | -2.37 | 0.62 |
| Item97 | -5.14 | 0.59 |
| Item98 | -1.58 | 0.51 |
| Item99 | -1.02 | 0.69 |
| Item100 | -2.14 | 0.83 |
| Item101 | 3.98 | 0.28 |
| Item102 | -3.33 | 0.37 |
| Item103 | -0.55 | 0.44 |
| Item104 | -2.24 | 0.79 |
| Item105 | -1.28 | 0.66 |
| Item106 | 6.24 | 0.14 |
| Item107 | -0.52 | 0.85 |
| Item108 | -2.02 | 1.46 |
| Item109 | -3.44 | 0.86 |
| Item110 | 3.06 | 0.26 |
| Item111 | -2.65 | 1.57 |
| Item112 | -2.92 | 0.85 |
| Item113 | 1.05 | 0.16 |
| Item114 | -5.80 | 0.70 |
| Item115 | -13.63 | 0.09 |
| Item116 | -2.63 | 0.30 |
| Item117 | -1.85 | 0.80 |
| Item118 | -2.37 | 0.72 |
| Item119 | 1.83 | 0.30 |
| Item120 | 0.54 | 0.51 |
| Item121 | 1.84 | 0.52 |
| Item122 | -2.16 | 0.90 |
| Item123 | -1.89 | 0.45 |
| Item124 | -1.88 | 1.04 |
| Item125 | -0.71 | 0.33 |
| Item126 | -3.22 | 0.97 |
| Item127 | -0.23 | 0.59 |
| Item128 | -1.85 | 0.52 |
| Item129 | 1.02 | 0.46 |
| Item130 | -2.40 | 0.56 |
| Item131 | -3.57 | 1.24 |
| Item132 | -1.61 | 0.64 |
| Item133 | -2.49 | 1.07 |
| Item134 | -3.17 | 0.59 |
| Item135 | -2.40 | 1.36 |
| Item136 | -3.31 | 0.83 |
| Item137 | -0.60 | 0.42 |
| Item138 | -1.93 | 1.45 |
| Item139 | -2.74 | 1.32 |
| Item140 | -2.27 | 0.71 |
| Item141 | -4.81 | 1.31 |
| Item142 | -2.56 | 0.91 |
| Item143 | -2.41 | 1.43 |
| Item144 | -4.21 | 0.79 |
| Item217 | -1.94 | 1.74 |
| Item218 | 1.15 | 0.51 |
| Item219 | -1.58 | 0.92 |
| Item220 | -1.96 | 0.78 |
| Item221 | -1.42 | 0.57 |
| Item222 | -1.79 | 0.74 |
| Item223 | 4.74 | 0.30 |
| Item224 | -0.84 | 0.66 |
| Item225 | -1.89 | 1.28 |
| Item226 | -1.77 | 0.87 |
| Item227 | -1.46 | 1.01 |
| Item228 | -1.99 | 1.38 |
| Item229 | -2.50 | 0.61 |
| Item230 | -1.83 | 1.29 |
| Item231 | -0.65 | 0.73 |
| Item232 | -1.64 | 1.02 |
| Item233 | -1.07 | 0.72 |
| Item234 | -0.06 | 0.73 |
| Item235 | -2.16 | 1.29 |
| Item236 | -0.23 | 1.01 |
| Item237 | -0.01 | 0.97 |
| Item238 | -1.74 | 1.10 |
| Item239 | 3.70 | 0.34 |
| Item240 | -2.59 | 1.45 |
| Item241 | 0.02 | 0.69 |
| Item242 | 0.23 | 0.70 |
| Item243 | -2.19 | 1.48 |
| Item244 | -2.22 | 0.71 |
| Item245 | 3.46 | 0.41 |
| Item246 | -1.74 | 1.25 |
| Item247 | 1.96 | 0.45 |
| Item248 | 2.95 | 0.50 |
| Item249 | -0.09 | 0.71 |
| Item250 | -1.98 | 0.89 |
| Item251 | -1.30 | 0.85 |
| Item252 | 4.10 | 0.42 |
| Item253 | -0.64 | 1.01 |
| Item254 | 3.72 | 0.32 |
| Item255 | 2.37 | 0.65 |
| Item256 | 2.51 | 0.40 |
| Item257 | -2.66 | 1.53 |
| Item258 | -2.65 | 1.15 |
| Item259 | -2.28 | 1.10 |
| Item260 | -1.48 | 0.97 |
| Item261 | -1.21 | 0.80 |
| Item262 | -2.28 | 1.17 |
| Item263 | -1.75 | 0.67 |
| Item264 | -3.03 | 1.21 |
| Item265 | -1.19 | 1.12 |
| Item266 | -1.59 | 0.72 |
| Item267 | -2.30 | 0.88 |
| Item268 | 1.30 | 0.55 |
| Item269 | -2.01 | 0.73 |
| Item270 | -1.24 | 0.79 |
| Item271 | -2.31 | 0.99 |
| Item272 | 2.82 | 0.48 |
| Item273 | 0.77 | 0.41 |
| Item274 | -2.14 | 1.29 |
| Item275 | -1.05 | 1.10 |
| Item276 | -2.60 | 1.00 |
| Item277 | -1.50 | 0.93 |
| Item278 | -2.16 | 0.88 |
| Item279 | -2.10 | 1.06 |
| Item280 | -1.71 | 1.39 |
| Item281 | -1.40 | 0.90 |
| Item282 | -2.54 | 1.07 |
| Item283 | -2.34 | 1.48 |
| Item284 | -3.14 | 1.33 |
| Item285 | -4.12 | 0.90 |
| Item286 | -2.36 | 1.86 |
| Item287 | -3.98 | 0.96 |
| Item288 | -2.06 | 1.62 |

Table S3

*Item parameters for items presenting same speaker identities (final short version of the Bangor Voice Matching Test)*

|  | Difficulty | Discrimination |
| --- | --- | --- |
| Item2 | -2.80 | 1.60 |
| Item4 | -2.62 | 1.45 |
| Item10 | -0.42 | 0.85 |
| Item15 | -2.51 | 1.32 |
| Item20 | -2.54 | 0.97 |
| Item23 | -5.42 | 0.83 |
| Item32 | -2.19 | 1.35 |
| Item35 | -2.07 | 1.01 |
| Item37 | -3.17 | 1.24 |
| Item38 | -2.79 | 1.51 |
| Item48 | -0.92 | 0.83 |
| Item49 | -1.93 | 1.21 |
| Item50 | -2.00 | 1.71 |
| Item53 | -2.02 | 1.01 |
| Item56 | 0.46 | 0.82 |
| Item57 | -2.96 | 1.31 |
| Item58 | -2.76 | 1.19 |
| Item62 | -2.87 | 2.13 |
| Item69 | -1.82 | 1.42 |
| Item72 | -5.01 | 1.06 |
| Item145 | -2.80 | 1.47 |
| Item155 | -1.62 | 1.09 |
| Item159 | -2.00 | 0.91 |
| Item173 | -2.36 | 0.87 |
| Item174 | -1.81 | 1.58 |
| Item178 | -2.16 | 0.95 |
| Item179 | -2.51 | 1.49 |
| Item180 | -2.24 | 1.32 |
| Item186 | -2.56 | 1.76 |
| Item188 | -2.18 | 1.02 |
| Item189 | -3.99 | 1.28 |
| Item193 | -1.41 | 1.00 |
| Item198 | -1.78 | 1.47 |
| Item201 | -1.88 | 2.51 |
| Item204 | -3.03 | 1.49 |
| Item206 | -0.05 | 0.68 |
| Item207 | -1.73 | 1.46 |
| Item208 | -2.66 | 1.33 |
| Item212 | -2.39 | 1.11 |
| Item215 | -2.78 | 2.47 |

Table S4

*Item parameters for items presenting different speaker identities (final short version of the Bangor Voice Matching Test)*

|  | Difficulty | Discrimination |
| --- | --- | --- |
| Item73 | -3.03 | 1.09 |
| Item75 | -1.89 | 0.96 |
| Item82 | -1.11 | 0.80 |
| Item87 | -1.87 | 1.01 |
| Item107 | -0.50 | 0.90 |
| Item108 | -2.02 | 1.48 |
| Item109 | -2.90 | 1.08 |
| Item111 | -2.50 | 1.76 |
| Item117 | -1.96 | 0.75 |
| Item122 | -2.08 | 0.96 |
| Item124 | -1.77 | 1.15 |
| Item126 | -2.85 | 1.15 |
| Item131 | -3.38 | 1.33 |
| Item133 | -2.56 | 1.04 |
| Item135 | -2.25 | 1.53 |
| Item138 | -1.89 | 1.53 |
| Item139 | -2.60 | 1.44 |
| Item141 | -4.15 | 1.61 |
| Item142 | -2.37 | 1.01 |
| Item143 | -2.16 | 1.77 |
| Item217 | -1.92 | 1.81 |
| Item225 | -1.88 | 1.30 |
| Item227 | -1.42 | 1.05 |
| Item228 | -2.01 | 1.36 |
| Item230 | -1.78 | 1.36 |
| Item232 | -1.52 | 1.14 |
| Item236 | -0.26 | 0.88 |
| Item237 | -0.01 | 0.90 |
| Item238 | -1.71 | 1.14 |
| Item243 | -2.15 | 1.54 |
| Item246 | -1.74 | 1.26 |
| Item251 | -1.39 | 0.78 |
| Item253 | -0.66 | 0.98 |
| Item260 | -1.60 | 0.88 |
| Item265 | -1.34 | 0.95 |
| Item275 | -1.01 | 1.19 |
| Item277 | -1.40 | 1.03 |
| Item280 | -1.62 | 1.55 |
| Item284 | -3.00 | 1.43 |
| Item288 | -1.99 | 1.75 |

Table S5

*Correlations of Bangor Voice Matching Test (BVMT) and Glasgow Voice Memory Test (GVMT) with all covariates*

|  | **Variables** | **1** | **2** | **3** | **4** | **5** | **6** |
| --- | --- | --- | --- | --- | --- | --- | --- |
|  |  |  |  |  |  |  |  |
| **1.** | **BVMT** | ̶ |  |  |  |  |  |
|  |  |  |  |  |  |  |  |
| **2.** | **GVMT: voice memory** | .23  (.009) | ̶ |  |  |  |  |
|  |  |  |  |  |  |  |  |
| **3.** | **GVMT: bell memory** | .25  (.005) | .12  (.184) | ̶ |  |  |  |
|  |  |  |  |  |  |  |  |
| **4.** | **Glasgow Face Matching Test** | .24  (.004) | .02  (.787) | .20  (.023) | ̶ |  |  |
|  |  |  |  |  |  |  |  |
| **5.** | **Profile of Music Perception Skills** | .37  (< .001) | .14  (.125) | .20  (.021) | .18  (.033) | ̶ |  |
|  |  |  |  |  |  |  |  |
| **6.** | **Digit Span** | .25  (.003) | -.01  (.905) | .06  (.509) | .10  (.224) | .20  (.013) | ̶ |

^Correlations are Pearson’s r. Numbers in parentheses represent p-values.^ *^N^* ^= 128 for all correlations involving the GVMT as the GVMT was only available for this subsample;^ *^N^*^= 149 for all other comparisons.^
